# From Their Perspective: Baby Boomers’ Health Information Searching Behaviors

**DOI:** 10.1093/geroni/igaa057.1362

**Published:** 2020-12-16

**Authors:** Tammy Walkner

**Affiliations:** University of Iowa, Solon, Iowa, United States

## Abstract

People frequently turn to the Internet for health information, especially as they get older. But searches and results can be influenced by many factors including the comfort level for using digital technology, input from social connections, and personal knowledge. The Baby Boom generation is changing the age structure of the U.S. toward an older age demographic. Despite the significance of this cohort, little is known about how they apply health information to manage personal health. This study focused on Baby Boomers to increase knowledge about their health information searching behavior, the role social support plays in that behavior, and to better understand their perceptions of healthy aging. Social cognitive theory (Bandura, 2004) was the framework to investigate micro-level factors that may influence Baby Boomers’ decisions about health information and whether they apply that information for healthy aging. Findings confirm Baby Boomers use online health information to advocate for specific treatments and more appropriate medications, to change their diet and exercise routines as well as to maintain their quality of life as they age. They also routinely rely on social connections when gathering information and implementing changes. Knowledge about Baby Boomers’ health information seeking behaviors has broad implications for health care professionals, web page designers, policy makers, and others interested in promoting healthy aging. Insight into older adults’ perceptions about using newer digital devices and their perseverance to learn new procedures like accessing electronic health records may spark changes in the communication between health care providers and patients of all abilities.

